# Detection and identification of huwentoxin-IV interacting proteins by biotin-avidin chemistry combined with mass spectrometry

**DOI:** 10.1186/1678-9199-20-18

**Published:** 2014-04-28

**Authors:** Hai Yu, Hui Liu, Yizhong Yan, Zhigui Duan, Xianchun Wang

**Affiliations:** 1 Key Laboratory of Protein Chemistry and Developmental Biology of Ministry of Education, College of Life Sciences, Hunan Normal University, Changsha 410081, People's Republic of China

**Keywords:** Biotin labeling, Huwentoxin-IV, Far-western blotting, Interacting protein, Mass spectrometry

## Abstract

**Background:**

Numerous spider toxins are of interest as tools for neurophysiological research or as lead molecules for the development of pharmaceuticals and insecticides. Direct detection and identification of the interacting proteins of a spider toxin are helpful for its action-mechanism analysis and practical application. The present study employed a combinative strategy for the analysis of interacting proteins of huwentoxin-IV (HWTX-IV), a peptidic neurotoxin from the venom of the spider *Selenocosmia huwena*.

**Results:**

HWTX-IV was first lightly labeled with biotin under the optimized mild experimental conditions and the toxin labeled with a single biotin group (monobiotinylated HWTX-IV) was demonstrated by electrophysiological experiments to retain its original bioactivity and was used in combination with far-western blotting to detect its interacting proteins. Comparative experiments indicated that some membrane proteins from rat neuromuscular junction preparations bind to monobiotinylated HWTX-IV after being transferred onto a PVDF membrane from the SDS-gel. With capillary high performance liquid chromatography-tandem mass spectrometry, several membrane proteins with which HWTX-IV potentially interacted were identified from the preparations and then bioinformatically analyzed.

**Conclusions:**

This work has provided not only a new insight into the action mechanism of HWTX-IV but also a reference technology for the relevant researches.

## Background

The venom of spiders is rich in neurotoxins that specifically act on the ion channels and ion channel-associated proteins of neurons, and therefore comprises a promising new source of molecules for the development of reagent tools for neurophysiological research and pharmaceuticals as well as insecticides [1-3]. For example, agatoxins from the venom of *Agelenopsis aperta* target three classes of ion channels, including transmitter-activated cation channels, voltage-activated sodium channels, and voltage-activated calcium channels. Of the agatoxins, polyamine α-agatoxins are use-dependent and their actions are synergized by the μ-agatoxins, which increase spontaneous transmitter release by modification of neuronal sodium channel gating. ω-Agatoxins produce long-lasting paralysis by acting in concert to block presynaptic calcium channels in nerve terminals. The α- and ω-agatoxins also block ion channels in the mammalian brain and have stimulated interest in drug development. The ω-agatoxins have become important pharmacological probes for characterization of vertebrate voltage-gated calcium channels. The μ-agatoxins are highly insect-selective and have potential as bioinsecticides [4]. In addition, α-latrotoxin – a high-molecular-weight protein toxin from the *Latrodectus* venoms that stimulates massive neurotransmitter release at vertebrate nerve terminals – has proven invaluable for studies of synaptic vesicle exocytosis [5]. Obviously, elucidation of the action targets of the toxins is of both theoretical and practical significance. Among the methods for the analysis, voltage-clamp and patch-clamp techniques have been most widely used, by which a number of spider toxins acting on the ion channels have been well characterized [6-8]. While such techniques investigate the action targets of a toxin primarily based on the changes in ion channel currents, some other techniques such as biotin labeling combined with far-western blotting and mass spectrometry provide more direct information by detecting and identifying the molecular targets of the toxin.

In this study we focused on the detection and identification of the interacting proteins with huwentoxin-IV (HWTX-IV), a peptidic neurotoxin with a molecular weight of 4.1 kDa isolated from the venom of the Chinese bird spider *Solenocosmia huwena*. HWTX-IV consists of 35 amino acid residues including 5 Lys residues and can completely block the nerve-muscle conduction of isolated phrenic nerve-hemidiaphragm preparations [9,10]. A whole-cell patch-clamp assay demonstrated that the toxin specifically inhibits the neuronal tetrodotoxin-sensitive (TTX-S) voltage-gated sodium channel with the IC_50_ value of 30 nM in adult rat dorsal root ganglion neurons, but has no significant effect on the tetrodotoxin-resistant (TTX-R) voltage-gated sodium channel. Additional research studies have suggested that HWTX-IV seems to be a site I toxin that affects the sodium channel by suppressing the peak sodium current and not altering the activation or inactivation kinetics, a mechanism quite similar to that of TTX. The three-dimensional structure of HWTX-IV has been determined and the comparison of the resulting structure with those of the others purified from the same species suggested that the positively charged residues of loop IV (residues 25-29, SRKTR), especially R26, may be crucial to its binding to the neuronal TTX-S voltage-gated sodium channel [11]. Although much work has been done to investigate the action mechanism of HWTX-IV, there are still many issues to be addressed. The present study employed a combinative strategy for the analysis of interacting proteins of HWTX-IV, in which the toxin was first lightly labeled with biotin under the optimized mild reaction conditions, and then the far-western blotting and capillary column high performance liquid chromatography-tandem mass spectrometry (CapLC-MS/MS) were used to detect and identify its interacting proteins. As a result, several membrane proteins with which HWTX-IV potentially interacted were identified from rat neuromuscular junction preparations and their relationship with the action of the toxin was discussed.

## Materials and methods

### Biotin labeling of HWTX-IV and labeled product separation

Biotin labeling of native HWTX-IV was performed according to the instructions of the manufacturers of biotinylation reagent (Pierce, USA) and previously reported methods [12,13]. HWTX-IV and biotinylation reagent Sulfo-NHS-Biotin were separately dissolved in 50 μL of 0.2 M phosphate buffered solution containing 0.15 M NaCl (pH 7.2) and then mixed at a Sulfo-NHS-Biotin to toxin ratio of 2:1. The labeling reaction was allowed to proceed on ice for two hours. The reaction solution was acidified with diluted TFA, followed by reversed-phase performance liquid chromatography (RP-HPLC) to remove the excess Sulfo-NHS-Biotin and desalt the sample. All the peptides were eluted from the reversed-phase column with a high concentration of ACN and the collected peptide mixture was analyzed with MALDI-TOF mass spectrometry to evaluate the labeling efficiency.

Cation exchange chromatography (CE-HPLC) on a Toyopearl® CM-650 M column (5 mm × 10 mm, Tosoh, Japan) was first employed to isolate the desired labeled product. The fractions eluted from the column with a NaCl gradient were separately desalted and further purified with RP-HPLC on a C4 column (300 Å, 4.6 mm × 250 mm, Phenomenex, USA), followed by MALDI-TOF mass spectrometric analysis. HWTX-IV labeled with one biotin group (monobiotinylated HWTX-IV) or two biotin groups (bisbiotinylated HWTX-IV), identified based on their molecular weights determined by the MALDI-TOF mass spectrometry, were separately collected for subsequent analyses.

### Bioactivity assay of biotinylated HWTX-IV

The bioactivity of monobiotinylated and bisbiotinylated HWTX-IV were measured using mouse isolated phrenic nerve-hemidiaphragm preparations according to a method described previously [14]. Briefly, the preparation was immersed in a Tyrode’s solution (NaCl 135.0, KC1 5.0, CaCl_2_ 2.0, MgCl_2_ 1.0, Na_2_HPO_4_ 0.08, glucose 1.0, pH 7.4) contained in a small plexiglass chamber, kept at 30-32°C and continuously bubbled with 95% O_2_/5% CO_2_. Proper electrical stimulation was applied to the phrenic nerve of the preparation with a suction electrode, and the resulting twitch responses of diaphragm muscle of the preparation were transformed into an electric signal and recorded with a signal processing system (Model BL420-S, China). The bioactivity of the biotinylated HWTX-IV was determined by comparing its effect with that of native HWTX-IV on the twitch responses of diaphragm muscle indirectly caused by electrical stimulation of the phrenic nerve at the same concentrations.

### Enrichment of diaphragm muscle plasma membrane

The enrichment of plasma membrane rich in neuromuscular junctions from adult Sprague-Dawley rats was performed according to previously described methods [15,16]. In brief, the center parts of the diaphragm muscles isolated from the rats anesthetized with ether and killed by decapitation were homogenized with a Dounce homogenator in a protease-inhibitor-containing homogenate buffer (250 mM sucrose, 10 mM Hepes, 1.0 mM EDTA, 100 mM PMSF, pH 7.4). After the homogenate was centrifuged at 600 g for ten minutes at 4°C, the resulting supernatant was recovered and the pellet was homogenized and centrifuged again under the same conditions. The obtained supernatants were combined and centrifuged at 100,000 g for two hours at 4°C (Beckman, Ti 70 rotor). The plasma membranes in the pellet were further enriched with a discontinuous sucrose density gradient (60%, 45%, 41% and 37%) centrifugation. After centrifugation at 240,000 g (SW-28 rotor, Hitachi, Japan) for four hours, the plasma membrane-enriched fraction at the interface between the 37% and 41% sucrose solutions was collected, followed by washing with 1.0 mM NaHCO_3_ and centrifugation at 100,000 g twice.

### Far-western blotting of HWTX-IV interacting proteins

The proteins in the plasma membrane-enriched fraction were extracted with 2% SDS and then separated by SDS-PAGE according to the method of Laemmli [17] under denaturing conditions in a 5% stacking gel and a 12% separation gel. The protein sample was loaded into several parallel gel wells each with 100 μg proteins. The SDS-PAGE was run at 15 mA on stacking gel and 25 mA on separating gel. After the electrophoresis, the gel was separated from the glass plates and the proteins resolved in three lanes were electrotransferred onto a PVDF membrane in a wet transfer system (Bio-Rad, USA) for two hours at 200 mA and 4°C. The PVDF membrane was cut into three strips that corresponded to the lanes 1 to 3, respectively.

To investigate the effect of renaturation on binding of the interacting proteins to monobiotinylated HWTX-IV, far-western blotting detections with and without a denaturation/renaturation step were conducted comparatively. Lane 1 was used to detect the binding of monobiotinylated HWTX-IV to its interacting proteins without undergoing the denaturation/renaturation treatment. Briefly, after being blocked with 5% (v/v) non-fat dry milk in TBST (150 mM NaCl, 0.1% Tween-20, 25 mM Tris, pH 7.5) for one hour at room temperature, lane 1 was incubated with monobiotinylated HWTX-IV in the same TBST solution for three hours at room temperature. After washing with TBST extensively, the membrane strip was incubated for two hours at room temperature with the HRP-conjugated avidin (10 μg/mL). After washing four times each for five minutes, the strip was exposed to Hyperfilm ECL (Amersham, USA) and recorded with a Chemidoc XRS^+^ system (Bio-Rad, USA).

Lane 2 was used to detect the effect of denaturation/renaturation treatment on the binding of interacting proteins to monobiotinylated HWTX-IV. The denaturation/renaturation treatment was performed according to the method described by Wu *et al.*[18]. Briefly, the membrane strip was incubated at room temperature for 30 minutes sequentially in AC buffer (1 mM DTT, 0.5 mM EDTA, 10% glycerol, 100 mM NaCl, 0.1% Tween-20, 2% skim milk, 20 mM Tris, pH 7.4) containing 6 M, 3 M or 1 M guanidine hydrochloride, followed by sequential incubation at 4°C for 30 minutes in the AC buffer containing 0.1 M guanidine hydrochxloride and at 4°C for one hour in the buffer free of guanidine hydrochloride. The rest of the treatment was the same as that for lane 1. Lane 3 was used as a control and treated the same as lane 1, except that the strip was not incubated with monobiotinylated HWTX-IV before incubation with HRP-conjugated avidin.

### In-gel digestion and identification of HWTX-IV interacting proteins

In-gel digestion and mass spectrometry-based identification of the proteins were performed according to a previous work [19]. The desired protein band determined according to the far-western blotting was excised from SDS/PAGE gel and subjected to successive destaining and dehydration with 50% acetonitrile. Before in-gel digestion, the proteins in gel slice were reduced with 10 mM DTT at 57°C for one hour and alkylated by 55 mM iodoacetamide in the dark at room temperature for 45 minutes *in situ*. Proteins were in-gel digested with trypsin overnight at 37°C. The resulting peptides were extracted and analyzed for protein identification by CapLC-MS/MS. Liquid chromatography was performed using an automated Agilent 1200 LC system (Agilent Technologies, Germany); and the peptides separated on the reversed-phase capillary column were directed into the electrospray ionization source of the coupled 3D high-capacity ion trap mass spectrometer (HCTultra™, Bruker Daltonics, Germany) for MS and MS/MS analyses. The acquired raw spectral data were processed; and Mascot-compatible MGF files were created and used to search against the international protein index (IPI) rat database (IPI_rat_v3.48). Proteins were identified on the basis of peptides whose ions scores exceeded the threshold, *p* < 0.05, which indicated identification at the 95% confidence level. The relevant information on the identified proteins was retrieved from the same database.

## Results

### Labeling extent determination and labeled product separation

**Figure 1:**
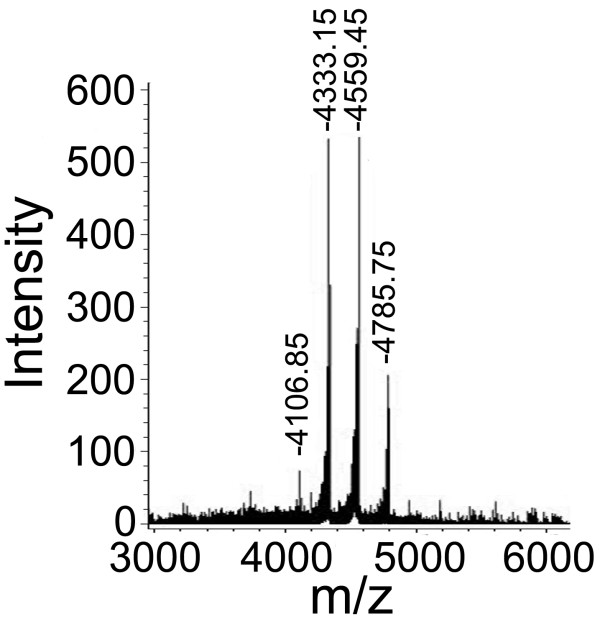
**Detection of the biotin labeling extent of HWTX-IV with MALDI-TOF mass spectrometry.** 4106.85 Da, unlabeled; 4333.15 Da, 4559.45 Da and 4785.75 Da, labeled with one, two or three biotin groups, respectively.

The purpose for the biotin labeling of HWTX-IV is to obtain the biotinylated products that have normal biological function and can be used to detect the interacting proteins of the toxin. Therefore, the mild biotinylation that decreases the risk of disrupting the bioactivity of the HWTX-IV is more desirable than the stronger. Biotin labeling extent is dependent on the experimental conditions, such as the ratio of biotin labeling reagent to the biological molecules, labeling reaction time and temperature, which enables us to control the biotinylation to a desired low level. We optimized the experimental conditions and found that when the ratio of biotin labeling reagent to the peptidic toxin was set at 2:1, the labeling reaction that proceeded on ice for two hours could achieve more desirable results. Under such mild experimental conditions, majority of HWTX-IV molecules were labeled with one, two or three biotin groups, with only a few being unlabeled, as determined by MALDI-TOF mass spectrometry. About 80% of the HWTX-IV molecules were labeled with one or two biotin groups, displaying respective molecular weights (M + H^+^) of 4333.15 Da and 4559.45 Da (Figure 1).

**Figure 2:**
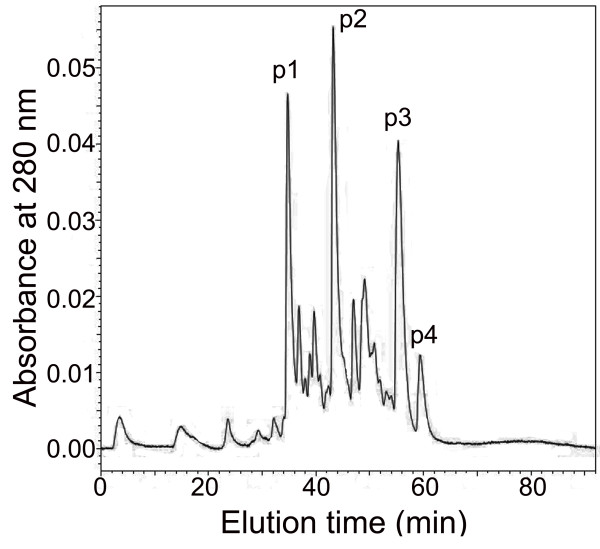
Cation exchange chromatographic profile of the labeled product mixture of HWTX-IV.

**Figure 3:**
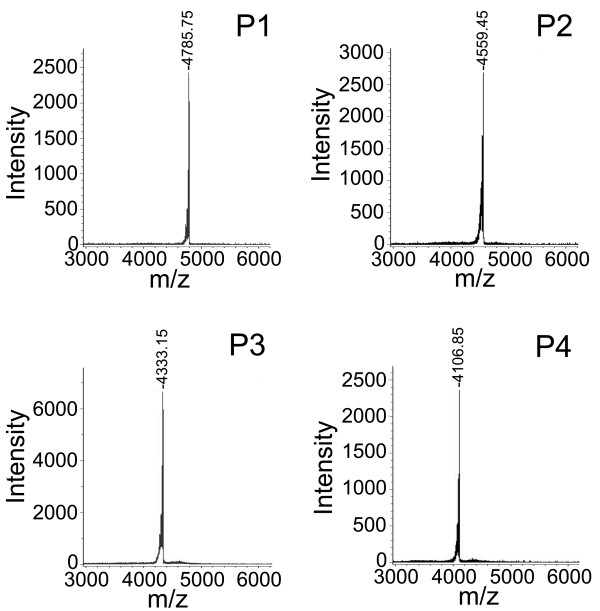
**MALDI-TOF mass spectrometric analysis of fractions P**_**1 **_**to P**_**4 **_**from CE-HPLC.**

In order to isolate desired biotin labeled HWTX-IV from the labeled product mixture, CE-HPLC and RP-HPLC were used. The resultant eluting curve of CE-HPLC (Figure 2) showed that the labeled product mixture was separated into four major peaks (P_1_-P_4_) and a series of minor peaks, suggesting that the mixture was efficiently separated based on the difference in the net charges. After the fractions P_1_-P_4_ were separately desalted and further purified by RP-HPLC, which displayed a single symmetric sharp peak in each chromatograms (not shown), MALDI-TOF mass spectrometry was employed to analyze the components in each fractions. The results indicated that the molecular weights (M + H^+^) of the components in fractions P_1_ to P_4_ were 4785.75 Da, 4559.45 Da, 4333.15 Da and 4106.85 Da, respectively, suggesting that fractions P_1_, P_2_ and P_3_ contained the HWTX-IV molecules that were labeled with three, two and one biotin groups, respectively, whereas P_4_ was composed of unlabeled HWTX-IV molecules (Figure 3). The MALDI-TOF mass spectra also revealed that there was only a single ion peak in each mass spectrum, indicating that HWTX-IV molecules with different labeling extents had been separated effectively.

### Bioactivity of biotinylated HWTX-IV

**Figure 4:**
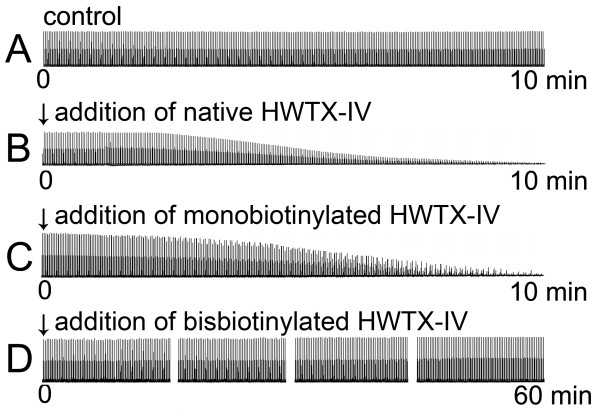
**Effect of HWTX-IV on the neuromuscular transmission in mouse isolated phrenic nerve-hemidiaphragm preparation. (A)** Control; **(B)** adding 2.4 × 10^-3^ μM unlabeled native HWTX-IV; **(C)** adding 2.4 × 10^-3^ μM monobiotinylated HWTX-IV; **(D)** adding 2.4 × 10^-3^ μM bisbiotinylated HWTX-IV.

Whether the biotin labeled HWTX-IV could retain its original bioactivity is critical for the subsequent experiments, and therefore the activity of the purified HWTX-IV labeled with one (monobioinylated HWTX-IV) and two biotin groups (bisbiotinylated HWTX-IV) was detected in mouse isolated phrenic nerve-hemidiaphragm preparations and compared with that of unlabeled native HWTX-IV. The result is shown in Figure 4.

In control experiments with the preparations immersed in Tyrode’s solution without addition of the toxin sample, there were no significant changes in the amplitude of nerve-evoked contraction of the diaphragm muscle in more than two hours (Figure 4 – A shows a segment of the muscle contraction curve diagram recorded). After the Tyrode’s solution was replaced with a Tyrode’s solution containing unlabeled native HWTX-IV at a concentration of 2.4 × 10^-3^ μM, the contraction amplitude of diaphragm began to decrease gradually after a latent period, and finally vanished completely in ten minutes (Figure 4 – B). This observation indicated that the transmission between phrenic nerve and hemidiaphragm was blocked, because the diaphragm contraction caused by direct stimulation at the muscle was not affected (the curve diagram not shown). At the same molar concentration, monobiotinylated HWTX-IV could also block the neuromuscular transmission in the preparations completely in ten minutes (Figure 4 – C), which meant that the neurotoxicity of monobiotinylated HWTX-IV was comparable to that of unlabeled native HWTX-IV. However, under the same experimental conditions the bisbiotinylated HWTX-IV had no significant effect on the neuromuscular transmission in 60 minutes (Figure 4 – D), suggesting that the HWTX-IV lost its activity due to the biotinylation by two biotin groups.

### Far-western blotting of HWTX-IV interacting proteins

**Figure 5:**
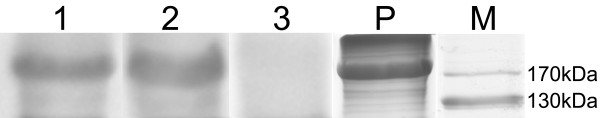
**Detection of HWTX-IV interacting proteins with far-western blotting.** Lane 1: without a denaturation/renaturation treatment before incubation with monobiotinylated HWTX-IV. Lane 2: with a denaturation/renaturation treatment before incubation with monobiotinylated HWTX-IV. Lane 3: incubation with HRP-avidin in the absence of monobiotinylated HWTX-IV. P: partial SDS-PAGE image showing the protein band corresponding to the far-western blotting-detected specific band on PVDF membrane. M: partial SDS-PAGE image showing protein molecular weight markers.

After the proteins extracted from the enriched diaphragm muscle plasma membrane were resolved by SDS-PAGE and electrotransferred onto the PVDF membrane, the interacting proteins of HWTX-IV were recognized by monobiotinylated HWTX-IV and visualized by HRP-conjugated avidin. For investigating the effect of denaturation/renaturation treatment on the HWTX-IV binding, comparative experiments were performed. The results showed that both lane 1 without a denaturation/renaturation treatement and lane 2 with such a treatment displayed strong binding bands at a molecular weight of about 170 kDa (Figure 5). The proteins in lane 1 could interact with monobiotinylated HWTX-IV even though they were not subjected to a denaturation/renaturation treatment before being incubated with monobiotinylated HWTX-IV, suggesting that they were renatured during the transfer and washing processes. Moreover, the interaction between monobiotinylated HWTX-IV and the proteins was specific, because the absence of monobiotinylated HWTX-IV led to the failure in the visualization of the binding band, as shown in lane 3 in Figure 5.

### Identification of HWTX-IV interacting proteins

According to the position of the specific band in the far-western blotting image, the counterpart protein band on the SDS-PAGE gel (lane P in Figure 5) was confirmed and excised, followed by in-gel digestion and CapLC-MS/MS analysis. By using the acquired spectral data to search against the international protein index (IPI) rat database, a batch of proteins were identified. After strict removal of both redundancy and the obviously contaminant proteins such as non-membrane proteins and some mitochondrial proteins, several membrane proteins or subunits were considered as the candidates that had the potential to interact with HWTX-IV (Table 1), including sodium/potassium-transporting ATPase subunit α-2, sarcoplasmic/endoplasmic reticulum calcium ATPase 1, and band 3 anion transport protein.

**Table 1:** The identified proteins that potentially interact with HWTX-IV

**Accession number**	**Description of proteins**	**Cellular location**	**Function**	**MW**	**Cov. (%)**
IPI00205693	Sodium/potassium-transporting ATPase subunit α-2	Cell membrane	Sodium/potassium transport	113457	51.57
IPI00213618	Sarcoplasmic/endoplasmic reticulum calcium ATPase 1	Endoplasmic reticulum membrane	Calcium transport	110707	56.54
IPI00231379	Band 3 anion transport protein	Cell membrane	Anion exchange	103392	21.57
IPI00207817	Ryanodine receptor 1	Membrane, Endoplasmic reticulum	Calcium transport	570767	23.16
IPI00231299	Voltage-dependent L-type calcium channel subunit α-1S	Membrane	Calcium transport	131428	18.50

**Figure 6:**
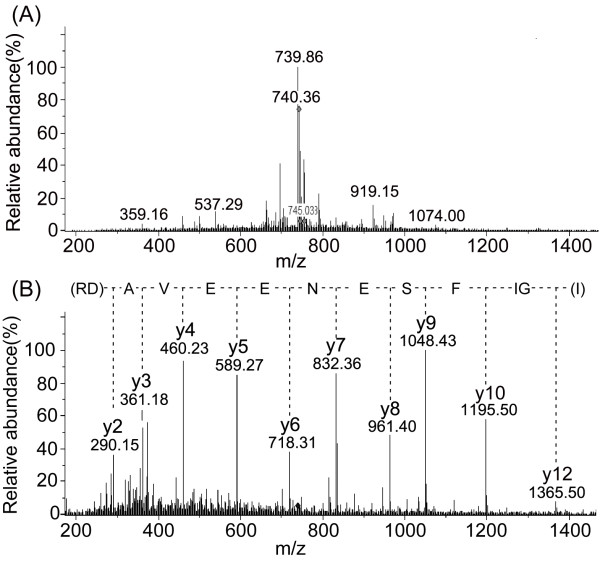
**Typical MS and MS/MS spectra of a peptide that was used for the identification of sarcoplasmic/endoplasmic reticulum calcium ATPase 1. (A)** MS survey scan during CapLC-MS/MS at time 34.9 minutes. **(B)** MS/MS spectrum of the precursor ion at m/z 740.36^2+^. The sequence was identified as IGIFSENEEVADR and the respective protein was sarcoplasmic/endoplasmic reticulum calcium ATPase 1.

All the proteins were unambiguously identified with high coverage (18.50-56.54%) and mass spectrometric data of high quality. Of these proteins, sarcoplasmic/endoplasmic reticulum calcium ATPase 1 presented the highest coverage while the typical MS and MS/MS spectra of a peptide that was used for identification of the protein is shown in Figure 6. In Figure 6 – B it can noted that the collision-induced dissociation of the doubly-charged peptide ion (740.36^2+^) in Figure 6 – A produced nearly complete y-series ions (y2-y12), which provided reliable sequence information for the identification of sarcoplasmic/endoplasmic reticulum calcium ATPase 1. In addition, all the identified proteins or subunits were involved in the across-membrane transport of ions including K^+^, Na^+^, Ca^2+^ and some anions. These data suggested that HWTX-IV might function by interacting with multiple kinds of ion channels in the membranes.

## Discussion

Biotin-avidin chemistry is commonly employed to detect and/or purify proteins and peptides because of their high mutual specificity. Biotin labeling is a key step in the application of the strategy [20]. Biotin labeling, also called biotinylation, is the process of attaching biotin to proteins and other macromolecules, which is most commonly achieved through chemical means, although enzymatic methods are also available [21]. Chemical methods not only provide greater flexibility in the type of biotinylation needed than enzymatic approaches but exhibit many other advantages such as easy operation and low cost [12]. The purpose of biotin labeling is to mark a protein of interest in such a way that the normal biological function of the protein is not significantly interrupted and can be used to detect and/or purify proteins in a biotin-avidin system. However, although the biotin molecule is small, biotinylation may interfere with normal functioning of the protein if the biotinylation extent is too large, which increases the risk of covalent attachment of the biotin group to the key amino acid residues or of disrupting the protein conformation. Therefore, the lower biotinylation extent is usually more desirable than the higher.

Biotin labeling extent is affected by experimental conditions including the ratio of biotinylation reagent to the protein, labeling reaction time and reaction temperature. On the other hand, it has been demonstrated that the solvent accessibility and interaction with adjacent amino acids of a K residue might contribute to the biotinylation extent of the residue [12]. That is to say, the reactivity of various K residues in HWTX-IV molecules is different. Under mild conditions (such as low ratio of biotinylation reagent to the protein and short reaction time), the K residues with higher reactivity would first be labeled, which provides an opportunity to control the labeling extent.

In the present study, HWTX-IV was biotin labeled at a lower level under the optimized mild experimental conditions, and MALDI TOF mass spectrometric analysis demonstrated that most of the HWTX-IV molecules had been labeled with one or two biotin groups. Biological mass spectrometry provided a convenient means for determining the biotinylation extent after a labeling reaction was performed and played important roles in optimizing the biotin-avidin assay system and ensuring reproducibility in the biotinylation process. Using CE-HPLC as well as RP-HPLC, the HWTX-IV molecules with different labeling extents were separated, from which we chose HWTX-IV labeled with one (monobiotinylated HWTX-IV) or two biotin groups (bisbiotinylated HWTX-IV) for bioactivity detection. Finally, we chose monobiotinylated HWTX-IV as the desired biotinylated product because it was demonstrated using mouse isolated phrenic nerve-hemidiaphragm preparations to retain the bioactivity comparable to that of the unlabeled native HWTX-IV. In contrast to monobiotinylated HWTX-IV, bisbiotinylated HWTX-IV lost its bioactivity, which was presumably due to the covalent attachment of the biotin group to the key residue of the HWTX-IV or the altered conformation of the toxin.

For efficient detection of the HWTX-IV interacting proteins in the membrane protein mixture, we first separated the mixture with SDS-PAGE in order to reduce the sample complexity and thus other proteins’ interferences. The resolved proteins were blotted onto the PVDF membrane and probed into with biotin labeled HWTX-IV combined with HRP-conjugated avidin. Obviously, the specificity of the interactions is crucial to the identification of interacting proteins. In view of this, we made comparative experiments with and without incubation with monobiotinylated HWTX-IV prior to the detection with HRP-conjugated avidin. The results showed that without monobiotinylated HWTX-IV, the HRP-conjugated avidin could not reveal the protein band, indicating that the detection had high specificity. Furthermore, when detecting the interacting partners of monobiotinylated HWTX-IV, far-western blotting was used, a technique requiring that at least the interacting domain of the target proteins for monobiotinylated HWTX-IV not be disrupted by SDS-PAGE and transfer or be able to refold on the membrane to form a native conformation containing an intact interaction site. Our comparative experiments demonstrated that, although the proteins underwent denaturation treatment prior to SDS-PAGE, they could be renatured after the SDS was eliminated during the transfer and washing processes, which is in agreement with the report of Wu *et al*. [18]. However, in the event that the protein is unable to refold to create an intact binding site, it is necessary to add a denaturation/renaturation step to the processes, which is typically accomplished by means of guanidine hydrochloride [18].

The application of high-sensitivity mass spectrometry with soft ionization enables the accurate identification of proteins in micro amounts. With the mass spectrometry-based strategy, we identified several membrane proteins or subunits from the protein band of interest that were able to interact with HWTX-IV or were highly homogenous with the interacting proteins of the toxin (Table 1). All the identified proteins were involved in the transport of the ions, especially Ca^2+^, K^+^, Na^+^ and some anions. Of the Ca^2+^ transporters, voltage-dependent calcium channels mediate the entry of Ca^2+^ into excitable cells and are also involved in a variety of calcium-dependent processes, including muscle contraction, hormone and neurotransmitter release. Voltage-dependent L-type calcium channel subunit α-1S produces L-type calcium currents whereas the calcium channels containing the α-1S subunit play an important role in excitation-contraction coupling in skeletal muscle [22]. Sarcoplasmic/endoplasmic reticulum calcium ATPase 1 and ryanodine receptor 1 are also Ca^2+^ transporters, and mediate the transport of Ca^2+^ across endoplasmic reticulum membrane, thereby involving muscular excitation/contraction [23,24]. These two proteins exist primarily in the endoplasmic reticulum membrane. However, it cannot be ruled out that the proteins are found in the plasma membrane because they may be transported from the endoplasmic reticulum membrane to plasma membrane via vesicle trafficking. Sodium/potassium-transporting ATPase subunit α-2 is the catalytic component of the active enzyme, which catalyzes the hydrolysis of ATP coupled with the exchange of sodium and potassium ions across the plasma membrane. In addition to the abovementioned cation transport proteins, the anion transport protein band 3 anion transport protein was also found to potentially interact with HWTX-IV. These data demonstrate that HWTX-IV can act on multiple ion channel proteins, especially the Ca^2+^ channels, and thus affect various physiological and biochemical processes in the target cells that are mediated by Ca^2+^, a typical secondary messenger, and some other ions. This finding is in agreement with the experimental results that the toxin could completely block the nerve-muscle conduction of isolated phrenic nerve-hemidiaphragm preparations, which involved multiple membrane proteins and/or ion channels [10].

## Conclusions

Herein we have detected the HWTX-IV interacting proteins in the preparation of neuron-muscle junctions using biotin-labeled HWTX-IV and far-western blotting, and demonstrated that the interaction between HWTX-IV and these proteins was specific, and that the electrotransfer and washing process were helpful for renaturation of the proteins. Based on mass spectrometry and bioinformatics, several membrane proteins mainly involving ion transport were demonstrated to have potentially interacted with the HWTX-IV. This work has provided not only a new insight into the action mechanism of HWTX-IV but also a reference technology for the relevant research studies.

### Ethics committee approval

All procedures conformed to the Guidelines of the National Institutes of Health Guide for the Care and Use of Laboratory Animals. The present study was approved by the Ethics Committee on the Use and Care of Animals of the Hunan Province, P. R. China.

## Abbreviations

HWTX-IV: Huwentoxin-IV; monobiotinylated HWTX-IV: HWTX-IV labeled by a single biotin group; bisbiotinylated HWTX-IV: HWTX-IV labeled by two biotin groups; CE-HPLC: Cation exchange high performance liquid chromatography; RP-HPLC: Reversed-phase high performance liquid chromatography; MALDI-TOF MS: Matrix-assisted laser desorption/ionization time-of-flight mass spectrometry; CapLC-MS/MS: Capillary liquid chromatography-tandem mass spectrometry.

## Competing interests

The authors declare that there are no competing interests.

## Authors’ contributions

HY, HL, YZY and XCW contributed to the design of the study. HY, HL, YZY and ZGD were responsible for collecting and analyzing samples. HY and XCW interpreted data and wrote the article. All authors read and approved the final manuscript.
